# Effects of smoking social cues on brand perception and smoking: an event-related potential study

**DOI:** 10.3389/fpsyt.2025.1518928

**Published:** 2025-04-23

**Authors:** Yiming Zhang, Zhan Wang, Fang Chen, Yuzhou Wang, Junyu Li

**Affiliations:** ^1^ Department of Marketing, China Tobacco Zhejiang Industrial Co., Ltd, Hangzhou, China; ^2^ School of Management, Zhejiang University, Hangzhou, China

**Keywords:** smoking social cues, brand perception, ERPs, N1 amplitudes, addictive behavior

## Abstract

**Introduction:**

Smokers’ dependence on tobacco products stems not only from substance addiction but also from social influences. While prior research has explored the impact of smoking action cues, it has largely overlooked smoking social cues and their role in shaping brand perception and smokers’ willingness to pay (WTP), leaving a gap in understanding their interaction. This study addresses the gap by analyzing event-related potentials (ERPs) and behavioral decisions in response to smoking social and action cues.

**Methods:**

Using a 2×2 design (social cues: present vs. absent; action cues: present vs. absent), we assessed brand perception, WTP, and N1 and P3 ERP amplitudes in 22 smokers (18 males, mean age 23.14 ± 1.60 years).

**Results:**

Results showed that smoking social cues increased brand perception and WTP while reducing N1 amplitudes, indicating that the presence of smoking social cues interfere with the processing of smoking action stimuli.

**Discussion:**

These findings highlights the importance of avoiding the simultaneous inclusion of social and action cues in anti-smoking advertisements, which also provide valuable insights for smoking cessation research.

## 1 Introduction and background

Smoking poses significant health risks, contributing to various diseases, including cancers and chronic disorders (1–3). Despite widespread awareness campaigns, many individuals continue to smoke, raising questions about the effectiveness of these initiatives in changing smokers’ cognitive perceptions (4). Research on the psychophysiological processes underlying smokers’ decision-making in smoking contexts remains limited. Understanding the purchasing decisions of addicted smokers is essential to identifying key influencing factors. Such insights can inform the development of effective policies to reduce smoking rates and promote healthier choices (5). Therefore, a comprehensive investigation into additional factors influencing smokers’ purchasing decisions and addictive behavior is urgently needed.

From a psychophysiological perspective, smoking is an addictive behavior influenced by both physiological cravings and cognitive processes (6–8). Smoking-related decisions can be divided into two stages: the purchasing stage and the smoking stage. Both are shaped by action cues and social cues. In the purchasing stage, smokers’ WTP for cigarettes is driven by physical cues such as brand appearance, name, imagery, and price, with particular sensitivity to new product types like low-tar or slim cigarettes (9, 10). Social cues act as external influences, encouraging smokers to emulate the behaviors and attitudes of those around them, thereby impacting both purchasing and smoking behaviors (7, 11). To effectively reduce cigarette consumption, interventions must go beyond addressing physiological cravings during the smoking stage (4, 6). Targeting purchasing behaviors and guiding smokers toward healthier alternatives are equally critical. This demands a comprehensive approach that accounts for the dynamic and often implicit cognitive processes underlying smoking decisions.

Given its sensitivity to dynamic cognitive processes, electroencephalogram (EEG) is a valuable tool for investigating the neuropsychological mechanisms and cognitive processes underlying smoking decisions (12–14). Event-related potential (ERP), an EEG-based technique, uses scalp electrodes to record brainwave changes, reflecting the brain’s responses to cognitive tasks. This method enables researchers to examine cognitive activity in detail. ERP is particularly effective for studying addictive behaviors like smoking, as it captures subtle and transient aspects of inhibitory control within cognitive processes. These nuances often go unnoticed in self-reported measures or behavioral assessments (14, 15).

ERPs studies of smoking behavior have identified distinct variations in N1 and P3 components. Existing literature suggests that exogenous components like N1 are influenced by the physical attributes of stimuli, while endogenous components such as P3 are linked to an individual’s internal mental state (16–18). Specifically, individuals with typical cognitive control exhibit higher N1 amplitudes and shorter P3 latencies in response to stop signals compared to individuals with impaired inhibitory control, reflecting enhanced attentional processing and faster inhibitory responses (19–23). Compared to non-smokers, smokers exhibit larger NoGo P3 amplitudes, indicating greater effort to inhibit responses (24–27). While much of this research has focused on clinical populations, it provides valuable insights into how cognitive control mechanisms vary under different task conditions. In summary, the N1 and P3 components in smokers differ from those of non-smokers in both amplitude and timing. These findings underscore the significance of N1 and P3 as markers of attentional processing and inhibitory control. However, existing studies seldom differentiate between various types of external stimuli, i.e. smoking action cues and smoking social cues. It remains unclear whether smoking social cues—such as images of multiple individuals engaged in smoking—enhance or interferes smokers’ processing of smoking-related stimuli (7, 26).

Previous research has emphasized the pivotal role of brand perception in shaping consumer purchasing decisions. Brand perception refers to how a brand is evaluated in comparison to similar products and can be understood from three perspectives: functional, symbolic, and experiential (28, 29). Positive brand perception—characterized by trust, quality, and emotional connection—has been shown to enhance consumers’ willingness to pay a premium and foster long-term brand loyalty (30, 31). Brand perception is influenced by both product attributes, such as objective cues related to the brand (32), and by consumers’ subjective motives, emotions, and intentions (33–35).

Neurophysiological studies further reveal that emotionally engaging brands trigger stronger neural responses in decision-making regions of the brain, which correlates with higher purchasing intentions (15, 36). Smaller P3 amplitudes are observed for brands with high awareness or well-matched brand extensions, suggesting that P3 is closely linked to the allocation of attentional resources (14, 37, 38). Additionally, a positive relationship between brand perception and emotion has been identified, with well-liked brands eliciting a more positive P3 component, indicating higher motivation levels (39). P3 also reflects the categorization process in working memory (40). Smokers who strongly identify with a brand are more influenced by changes in tobacco product characteristics, such as packaging (41–43). Despite abundant neurophysiological research on brand perception (36–43), there is limited literature addressing its impact in smoking contexts. We argue that brand perception plays a crucial role in both tobacco product purchasing and smoking behaviors.

According to cue utilization theory (44), the objective attributes of brand perception can be categorized into internal and external cues. Internal cues pertain to a product’s functional value, such as quality, size, and color. In contrast, external cues refer to information unrelated to the product’s inherent functionality, including brand name, reputation, price, and advertising imagery (9, 10). Compared to internal cues, external cues are more accessible and noticeable to consumers, making them more likely to attract attention and shape cognitive evaluations (44, 45).

In addition to product-related proximal stimuli, which include internal and external smoking-related cues that influence smokers’ responsiveness to tobacco products, distal stimuli may also have significant effects (46–49). Proximal cues are directly linked to smoking itself, such as cigarette products and packaging (50, 51), while distal cues are indirectly related to smoking, including social cues from environments like contextual or geographic factors (52, 53).

Many smokers experience cravings to smoke not solely due to nicotine dependence, but also because of the desire to engage in social activities (54, 55). Research has found out that smoking social cues such as the presence of others who are smoking can act as triggers that intensify the craving for smoking, as smokers tend to align their behavior with others in a social environment (56–60). The social rewards associated with smoking may lead smokers to perceive higher brand value, resulting in more pronounced cue reactivity and increased smoking behaviors (61–63).

Additionally, smokers react more impulsively to cues depicting human interaction with cigarettes than to cues featuring smoking actions alone (41, 64, 65). However, most neurophysiological research on smoking has primarily focused on smoking action cues, such as comparing the effects of cigarette images versus images of people smoking. Few studies have examined the differences between smoking action cues and smoking social cues in influencing smokers’ purchase intentions for tobacco products (7, 64). This study incorporates both smoking action cues and social cues to examine their interacting effect. The goal is to deepen our understanding of how social factors contribute to brand perceptions among smokers and to investigate the neurophysiological mechanisms underlying increased smoking behavior in social smoking contexts.

Smoking action cues elicit larger N1 amplitudes than non-smoking cues, suggesting heightened attentional salience for smokers (7, 27). Based on this, we hypothesize that smokers show enhanced neural attention to smoking cues due to the motivational salience of these stimuli. In contrast, social cues generate smaller N1 amplitudes compared to non-social cues. This may reflect reduced attentional discrimination due to the cognitive load or distraction introduced by social elements (22). Therefore, we hypothesize that the presence of social cues weakens attentional focus, potentially impairing smokers’ ability to process and differentiate salient stimuli in social contexts. Building on these findings, we further hypothesize that when smoking cues are presented in social contexts, the interaction between the salience of smoking action cues and the distraction from social elements may modulate neural attention responses. Specifically, social contexts could diminish smokers’ neural responses to smoking cues. Thus, we propose the following hypothesis:

H1a: Smoking action cues will enhance brand affinity compared to conditions without action cues.
H1b: Smoking action cues will enhance brand value compared to conditions without action cues.
H1c: Smoking action cues will increase willingness to pay compared to conditions without action cues.
H2a: Smoking social cues will enhance brand affinity compared to conditions without social cues.
H2b: Smoking social cues will enhance brand value compared to conditions without social cues.
H2c: Smoking social cues will increase willingness to pay compared to conditions without social cues.
H3a: Smoking action cues will elicit higher P3 amplitudes compared to conditions without action cues.
H3b: Smoking action cues will elicit higher N1 amplitudes compared to conditions without action cues.
H4a: Smoking social cues will elicit lower P3 amplitudes compared to conditions without social cues.
H4b: Smoking social cues will elicit lower N1 amplitudes compared to conditions without social cues.

In summary, this study aims to investigate the influence of smoking action cues and smoking social cues on brand perception of cigarettes, using ERP and behavioral decision experiments. The key components of this research include: (1) ERP characteristics of individuals in response to smoking action cues and social cues; (2) Brand affinity, brand value, and WTP for cigarettes in response to smoking action cues and social cues; (3) Analysis of ERP and behavioral data to examine the individual and interactive effects of smoking action cues and social cues.

## 2 Methods

### 2.1 Participants

We recruited 22 university student smokers (18 males, mean age 23.14 ± 1.60 years) through online social forums and WeChat groups between March 3 and April 1, 2023. All participants met the World Health Organization (WHO) 1997 criteria for “regular smokers” or “occasional smokers,” defined as smoking at least four times per week (66). All participants were daily smokers, had normal or corrected vision, and were right-handed. Exclusion criteria included: (1) the presence of any current physical or psychiatric illness; and (2) enrollment in any treatment or program. To minimize variability in smoking frequency and amount, we carefully controlled the daily smoking quantity of participants during recruitment, ensuring consistency across individuals. The average daily smoking quantity for participants was 10.3 cigarettes per day (SD ± 3.5 cigarettes). To avoid potential floor or ceiling effects from nicotine cravings during the task, participants were instructed to refrain from smoking for two hours prior to the experiment (47, 67). The EEG experiment was conducted from April 24 to 26. Prior to the study, each participant provided written informed consent for the EEG experiment. The study was approved by the Zhejiang University Internal Review Board of the Neuromanagement Lab.

### 2.2 Background cue

**Figure 1:**
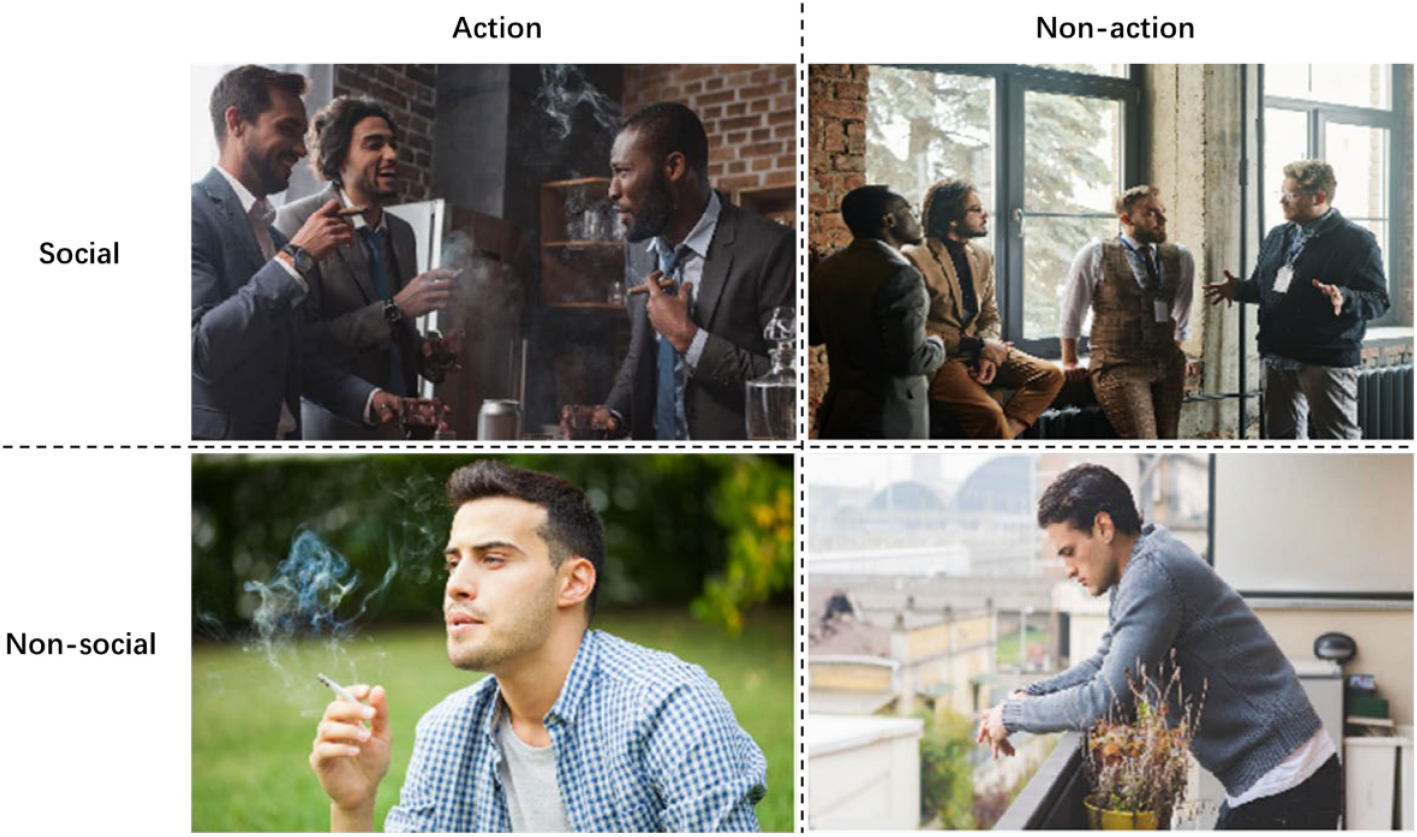
Cue stimuli. Actions of individuals were kept as consistent as possible and positioned at the center of the images. Copyright 2025 by Freepik Company S.L. Reprinted with permission.

he ERP experiments were designed using a 2 (action vs. non-action) × 2 (social vs. non-social) paradigm. Participants were tasked with evaluating cigarette brands after being exposed to various stimuli in different contexts, as depicted in the images presented in **Figure 1**. Smoking social cues were represented by images featuring multiple individuals, while smoking action cues showed individual(s) actively smoking. In the non-social condition, images depicted a single person, and in the non-action condition, individual(s) were shown not smoking. This categorization was designed to enhance our understanding of the motivations and characteristics underlying smoking behavior, isolating the effects of smoking cues on brand perception and WTP.

To minimize potential variations in brain activity due to differences in eye movement and gaze patterns, we ensured that the actions of individuals in the images were as consistent as possible, with subjects positioned centrally in the images. The images were carefully selected to focus solely on smoking-related action and social cues, minimizing any confounding effects related to cultural or ethnic associations. To further ensure neutrality, we used images of individuals with Western attributes, reducing the likelihood of prior familiarity among our participants (Chinese students) with the experimental materials.

**Figure 2:**
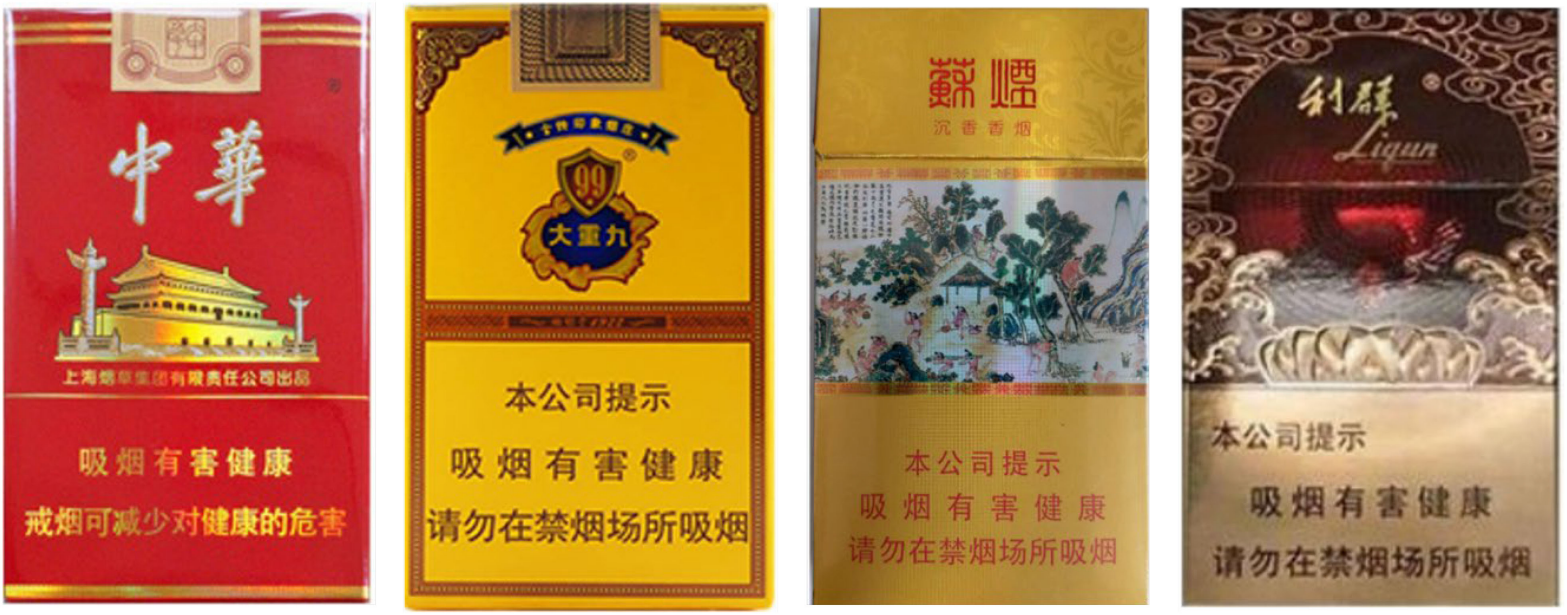
Brand Stimuli. 4 of the 32 brand images were shown as an example. No significant differences of familiarity and acceptance were observed between these 32 cigarette brand images.

Additionally, to control for participants’ potential pre-existing preferences toward different cigarette brands, we selected two images for each of 16 cigarette brands with similar market prices, based on the CNPP Brand Ranking of 16 cigarette brands (https://www.cnpp.cn/china/list_1353.html, see **Figure 2**). A pretest conducted with a separate group of 11 smokers assessed the familiarity, acceptance, brand affinity, brand value, and WTP for these 32 cigarette brand images. No significant differences were observed.

### 2.3 Procedure

**Figure 3:**
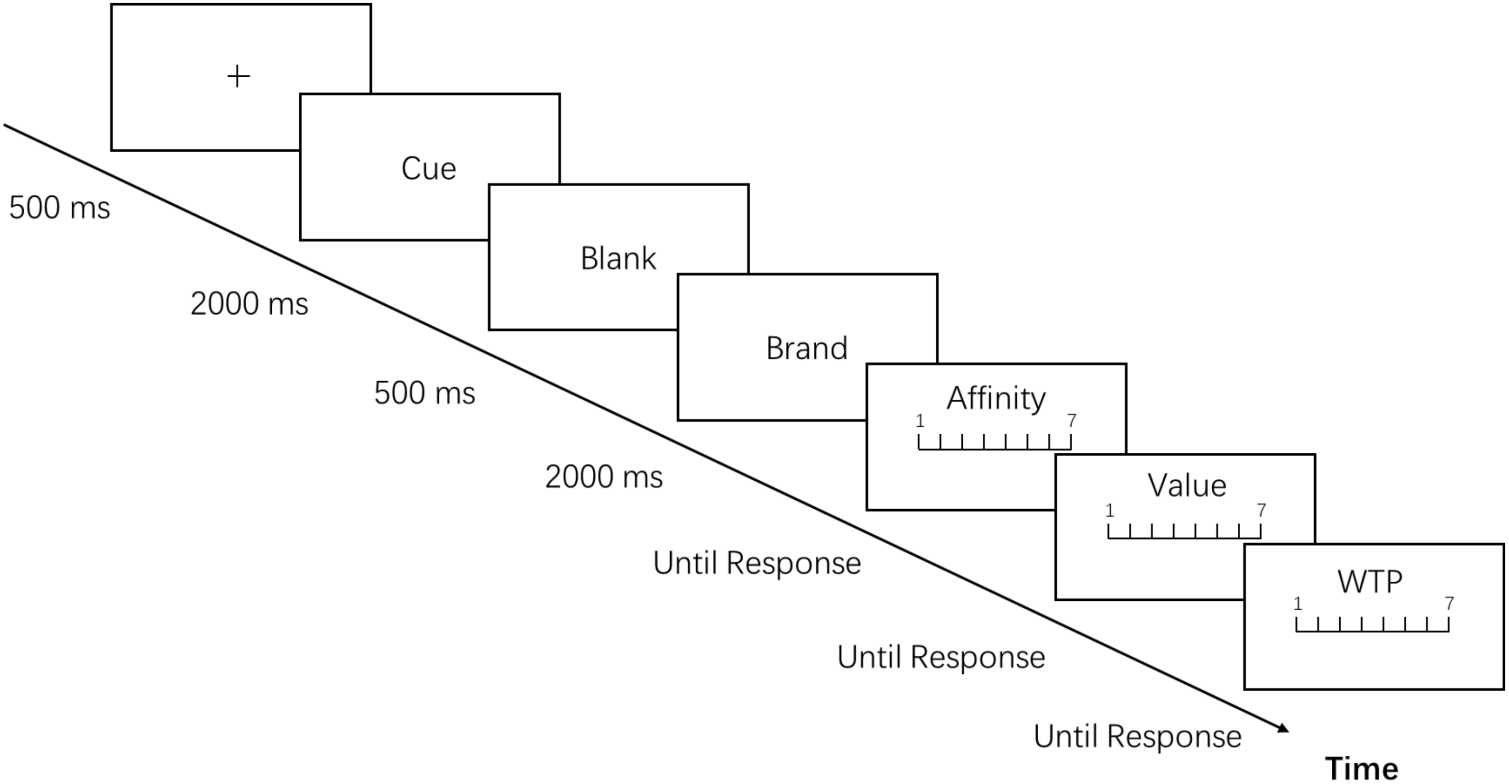
Experiment procedure. The timeline of one trial is shown. A fixation cross at the screen center (500 ms) was immediately followed by a cue (2,000 ms), a blank (500 ms), a brand image (2,000 ms) and three rating tasks (68, 69). The next trial started after 500 ~700 ms.

Participants were individually escorted into the laboratory and seated 70 cm away from the monitor. The experimental stimuli were centrally presented on the screen and controlled using E-Prime 2.0 software. All stimuli were displayed against a white background, and participants were provided with a keyboard to rate brand affinity, brand value and WTP. The experimental trial design is outlined in **Figure 3**. Each trial began with a “+” symbol displayed at the center of the screen, serving as a visual fixation point for 500 milliseconds (15). After this, images from the four sets of stimulus cues were randomly presented for 2000 milliseconds (68). A blank screen appeared for 500 milliseconds as a buffer before the next phase.

Subsequently, a cigarette brand image was displayed for 2000 milliseconds (69), followed by the first brand perception question on brand affinity: “How much do you like this brand?” Participants responded using a seven-point rating scale (with keys 1-7 representing “very bad” to “very good”). This was followed by the second question on brand value: “To what extent do you think this brand is valuable?” After the second response, the third question was presented: “To what extent are you willing to pay for this brand?” Between each response, a random interval of 500-700 milliseconds occurred before the next trial began.

The experiment followed a 2 (action vs. non-action) × 2 (social vs. non-social) × 2 images × 16 brands design, resulting in a total of 128 trials, randomly assigned into 4 blocks. Each participant started with one of the four blocks and experienced all trials in a random order. The experiment took approximately 20 minutes to complete. Given the passive observation design, the experiment was intended to elicit N1 and explore P3 components, though the lack of a response requirement might limit the capture of the latter. Participants were compensated for their participation after completing the experiment. All experimental methods followed relevant guidelines and regulations.

### 2.4 Electroencephalographic recording and analysis

EEG data were recorded using a 64-channel Neuroscan amplifier and Scan 4.5 software, following established guidelines (68). Vertical and horizontal eye movements were simultaneously recorded. The setup used average referencing, alternating current (AC) collection, with a sampling rate of 500Hz per channel and a band-pass filter range of 0.01-100Hz. Scalp impedance was kept below 10KΩ. The EEG data were processed using E-Prime 2.0 and MATLAB software. The data processing steps were as follows: initially, redundant electrodes (VEO, HEO, CB1, CB2, M1) were removed (70). The data were then filtered with a band-pass range of 0.1-30 Hz, and M2 was used for re-referencing before being removed. Independent component analysis (ICA) was performed to eliminate artifacts associated with eye movements and blinks. The data were then segmented into epochs of 1000 ms (from -200 to 800 ms) (68). The period from -200 to 0 ms was used as the pre-stimulus baseline for correction, and fluctuations exceeding ±100 μV were excluded.

This study specifically examined the maximum peak amplitudes of the N1 and P3 components evoked by the four types of cue stimuli. The most negative peak for N1 occurred approximately 100-200 ms after stimulus onset, while the most positive peak for P3 appeared around 250-400 ms (15, 27). The peak amplitudes of N1 and P3 within the respective time windows were automatically measured for each of the four stimulus conditions. Finally, the EEG data for the four types of stimuli were categorized by context and averaged.

### 2.5 Statistical analysis

Data analysis and statistical processing were conducted using SPSS 27 software. The analysis primarily involved mean comparisons, repeated measures ANOVA, and paired-samples t-tests. Cue types served as within-subject factors, and F-ratios associated with repeated-measures factors were corrected using the Greenhouse-Geisser procedure. These methods were applied to analyze the behavioral response data.

The ERP variables consisted of the mean peak amplitudes of the N1 and P3 components recorded at specific electrodes. For the analysis, a subset of electrodes was selected based on their established relevance to the ERP components of interest. N1 amplitudes were analyzed at frontal and central electrodes (F3, Fz, F4, FC3, FCz, FC4, C3, Cz, C4), where this component is typically maximal and reflects early attentional processes (14). P3 amplitudes were analyzed at parietal and centroparietal electrodes (P3, Pz, P4, CP3, CPz, CP4), which reflect attentional allocation and motivational salience (71). This targeted selection reduced redundancy and enhanced result interpretability.

The normality of N1 and P3 amplitudes was assessed using the Shapiro-Wilk test and visual inspection (e.g., Q-Q plots). No significant deviations from normality were observed (p > 0.05 for all conditions), supporting the use of parametric statistical methods. For cross-validation, non-parametric alternatives (e.g., Wilcoxon signed-rank tests) were conducted where necessary, yielding consistent results. In addition to ERP variables, the normality of behavioral variables, including brand affinity, brand value, and WTP, were also tested and confirmed.

## 3 Results

### 3.1 Behavioral results

As shown in **Table 1**, descriptive statistical analysis is conducted by comparing participants’ ratings of cigarette brand perception and WTP across the four conditions.

**Table 1:** Cues effects on brand perception and WTP.

Condition	Brand affinity Mean (SD)	Brand Value Mean (SD)	WTP Mean (SD)
Action * Social	4.99 (1.60)	5.12 (1.71)	4.54 (1.60)
Action * Non-social	4.84 (1.67)	4.80 (1.78)	4.39 (1.67)
Non-action * Social	4.89 (1.65)	4.89 (1.82)	4.31 (1.65)
Non-action * Non-social	4.63 (1.80)	4.58 (1.93)	4.09 (1.80)

All 22 participants completed 128 trials divided into 4 blocks. Hence, each condition comprised an average of 32 trials and the average across the 22 participants.

**Figure 4:**
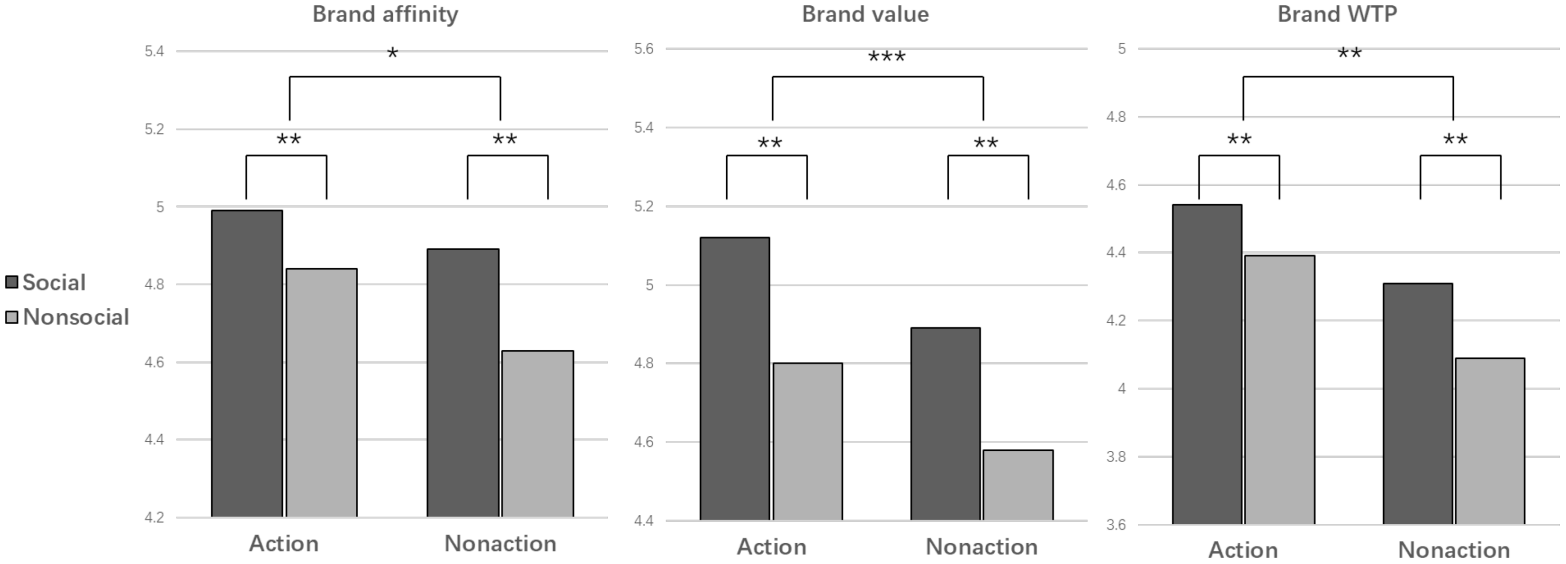
Mean of the brand affinity, brand value and brand WTP rating in four conditions. ***p<0.001, **p<0.01, *p<0.05.

The results of the ANOVA on behavioral variables are presented in **Table 2** and **Figure 4**. Firstly, a significant effect of the action cue was observed. Images with an action cue significantly enhanced brand affinity, brand value, and WTP (*F* (1,20) =5.664, *p*=0.018, *η_p_^2^ =* 0.01; *F* (1,20) =19.595, *p*<0.001, *η_p_^2^ =* 0.033; *F* (1,20) =7.141, *p*=0.008, *η_p_^2^ =* 0.033). Secondly, the effect of social cue on brand perception and WTP was also significant. Stimuli with a social cue significantly increased brand affinity (*F* (1,20) =8.96, *p*=0.003, *η_p_^2^ =* 0.015) and brand value (*F* (1,20) =9.275, *p*=0.002, *η_p_^2^ =* 0.016), consequently enhancing WTP (*F* (1,20) =14.272, *p*=0.002, *η_p_^2^ =* 0.016). However, the interaction between action cue and social cue did not have a significant effect on brand perception and WTP (*F* (1,20) =0.609, *p*=0.435, *η_p_^2^ =* 0.001; *F* (1,20) =0, *p*=1, *η_p_^2^ =* 0; *F* (1,20) =0.24, *p*=0.625, *η_p_^2^ =* 0.000).

**Table 2:** Results of repeated measures ANOVA.

Variables	Smoking Cues	Mean (SD)	*F*	*p*	*η_p_^2^*
Brand affinity	Action vs Non-Action	4.91 (1.63) vs 4.76 (1.73)	5.664	0.018	0.01
	Social vs Non-Social	4.94 (1.63) vs 4.73 (1.74)	8.96	0.003	0.015
Brand value	Action vs Non-Action	4.96 (1.75) vs 4.77 (1.90)	19.595	<0.001	0.033
	Social vs Non-Social	5.01 (1.77) vs 4.69 (1.86)	9.275	0.002	0.016
Brand WTP	Action vs Non-Action	4.46 (1.73) vs 4.20 (1.82)	7.141	0.008	0.033
	Social vs Non-Social	4.42 (1.76) vs 4.24 (1.80)	14.272	0.002	0.016

### 3.2 ERP results

**Figure 5:**
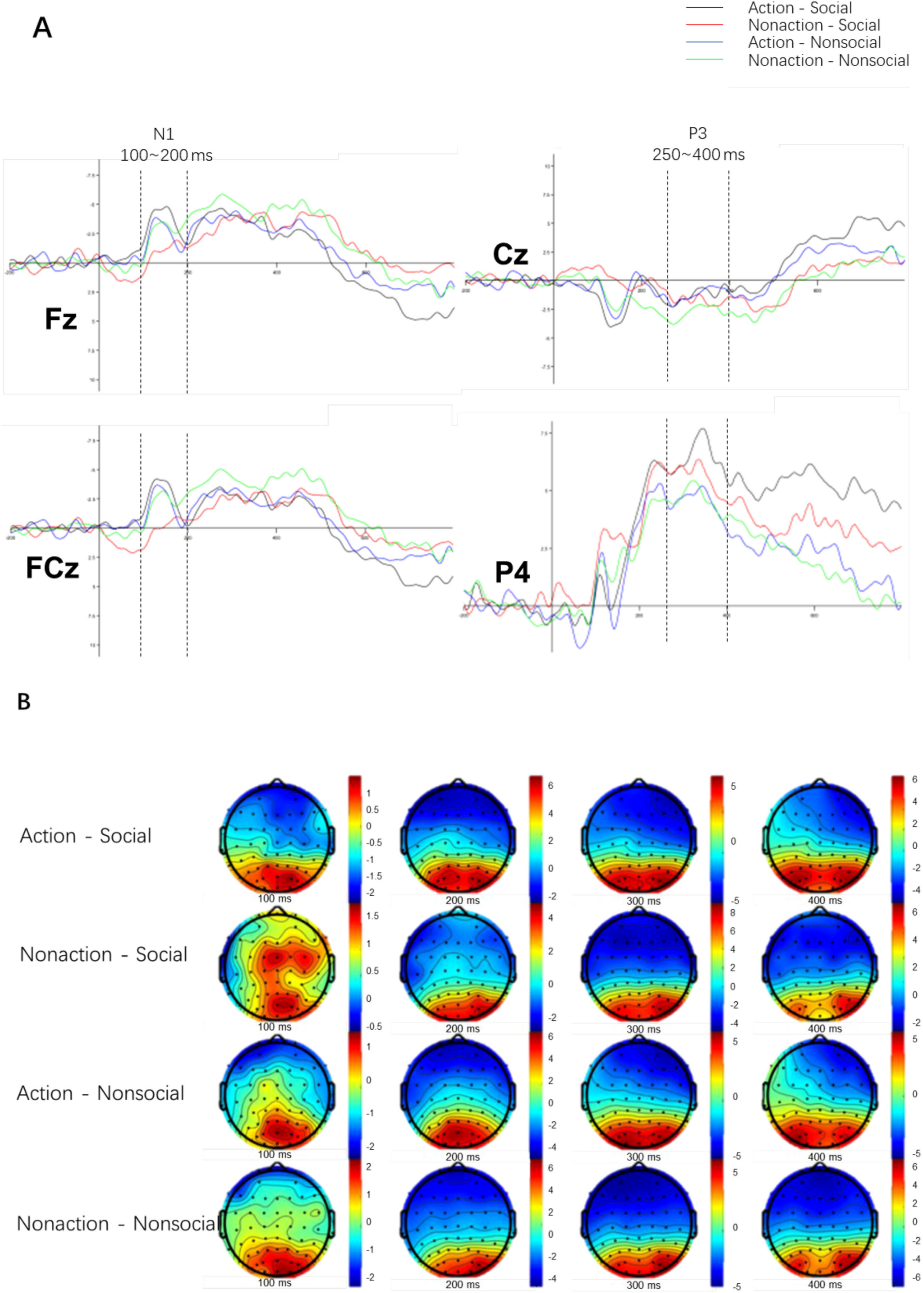
**(A)** Grand average waveforms of N1 and P3 components for selected electrode sites. The x-axis represents time (ms), with 0 indicating stimulus onset, and the y-axis represents amplitude (μV). The waveforms compare smoking and non-smoking cues under social and non-social conditions. **(B)** Topographical distribution of N1 (100–200 ms) and P3 (250–400 ms) amplitudes, showing the spatial localization of these components across the scalp. Warmer colors represent higher positive amplitudes, and cooler colors represent higher negative amplitudes. Electrodes used for statistical analysis (e.g., Fz, Cz, Pz) are highlighted.

**Figure 5** and **Table 3** show the ERP amplitude results for smokers across the four conditions: Action * Social, Action * Non-social, Non-action * Social, and Non-action * Non-social. As shown in **Figure 5A**, the average N1 amplitude under the smoking action condition (black and blue curves) was significantly higher than under the non-action condition (red and green curves). **Figure 5B** demonstrates that the brain topography around 100 ms significantly differs across the conditions.

**Table 3:** Repeated measures ANOVA of N1 and P3 amplitude by condition.

ERP component	Cues	Mean (SD)	*F*	*p*	*η_p_^2^*
N1	Action vs Non-action	-7.21 (1.33) vs -5.92 (1.31)	39.014	<0.001	0.629
	Social vs Non-social	-5.47 (1.24) vs -7.79 (1.23)	4.422	0.047	0.161
P3	Action vs Non-action	1.99 (3.44) vs 1.11 (2.99)	1.85	0.197	0.125
	Social vs Non-social	2.06 (3.35) vs 1.04 (3.07)	0.408	0.534	0.03

For N1 component, the ANOVA revealed a significant main effect of smoking action cue (*F* (1,20) =39.014, *p*<0.001, *η_p_^2^ =* 0.629) and smoking social cue (*F* (1,20) =4.422, *p*=0.047, *η_p_^2^ =* 0.161). Furthermore, there is a significant interaction effect between smoking action cue and smoking social cue (*F* (1,20) =6.535, *p*=0.018). Specifically, smokers exhibited a larger N1 amplitude following exposure to smoking action cue (M = -7.21) comparing to nonaction cue (M = -5.92). However, in the presence of social cues, a reduced N1 amplitude was observed after exposure to smoking social cue (M = -5.47) comparing to nonsocial cue (M = -7.79).

As for P3 component, the ANOVA results indicate that the main effect of smoking action cues is not significant (*F* (1,20) =1.85, *p*=0.197, *η_p_^2^ =* 0.125), neither is the main effect of smoking social cues (*F* (1,20) =0.408, *p*=0.534, *η_p_^2^ =* 0.03), and the interaction effect between smoking cues and smoking social cues is not significant (*F* (1,20) = 0.044, *p*=0.838). In other words, among smokers, there were no observed differences in the P3 component when exposed to action cues and social cues.

## 4 Discussion

This study employed ERP to investigate the impact of smoking social and action cues on the perception of cigarette brand among smokers, aiming to establish a connection between external cues, ERP, brand perception and finally smokers’ WTP.

The results revealed that, firstly, from a behavioral perspective, smokers exhibited higher brand perception and WTP for cigarette brands when exposed to action and social cue stimuli, supporting hypotheses H1a–H1c and H2a–H2c. This aligns with previous research findings where action and social cues were found to affect addicts’ craving for the product or brand. In our study, we verified that action cues can cause smokers to experience heightened craving for cigarette products, demonstrated by enhanced brand affinity and perceived value, resulting in a higher WTP. We also observed that the effect of social cues is independent of action cues and acts as an additional boost to smokers’ brand perception, further increasing WTP. The independent effects of two types of cues align with the categorization of internal and external cues in cue utilization theory (44, 45). Action cue stimuli are based on external cues, driven by the physical satisfaction derived from the components present in the cigarette itself, while social cues operate through internal cues, where the behavioral influence stems from the social interactions and exchanges that occur during smoking, providing a form of social reward. Research has shown that social cues are more effective in stimulating smoking behavior compared to action cues, as social engagement activates mechanisms like imitation and consumes self-regulatory cognitive resources (38, 41, 65). Our study provides behavioral experimental support for this line of research.

From a neurophysiological perspective, our study found that smokers exhibited larger N1 amplitudes in response to smoking action cues but smaller N1 amplitudes in response to smoking social cues, supporting hypotheses H3b and H4b. The larger N1 amplitudes observed in the smoking cue condition, compared to non-smoking cues, are consistent with prior research, indicating that smoking-related stimuli are highly salient and effectively capture smokers’ attention (7, 27). This attentional bias highlights the motivational relevance of smoking cues for individuals with nicotine dependence, reinforcing their role as potent triggers for smoking behavior. Conversely, the smaller N1 amplitudes observed in the social cue condition, compared to non-social cues, suggest that social elements reduce attentional discrimination. This diminished response may be attributed to the cognitive load or distractions introduced by social contexts, which compete for neural resources (22). Importantly, when smoking cues are presented within a social context, the interaction between these factors appears to weaken the attentional salience of smoking-related stimuli. This finding suggests that social contexts may attenuate smokers’ attentional biases toward smoking cues, with the cognitive demands or distractions of social interactions potentially interfering with the processing of addiction-related stimuli. This neurophysiological finding aligns with our behavioral results, where significantly lower willingness to pay (WTP) was observed when both smoking social cues and smoking action cues were present. This supports our initial hypothesis that social context can modulate smokers’ responses to smoking cues.

Regarding the P3 component, although the amplitudes under smoking action cues and smoking social cues were higher than those in the no-cue condition, the differences were not statistically significant. Therefore, hypotheses H3a and H4a were not supported. This lack of significant difference might be attributed to the differences in the experimental paradigm. In previous studies comparing smokers and non-smokers, smokers typically exhibit larger P3 amplitudes in response to cue stimulation, indicating that their inhibitory control demands higher cognitive resources (26, 27). However, in our experiment, where all participants were smokers, no significant differences were found in the P3 component between action and social cue conditions. This suggests that smoking cues may have become routine or automatic stimuli within the internal cognitive processes of smokers, and as such, they do not necessitate additional cognitive resource allocation (72). Additionally, the passive observation design employed in this study, where participants were only required to rate brand perception without actively engaging in inhibitory responses as they would in a NoGo task, may have further attenuated the significance of any P3 amplitude differences. Together, these may have contributed to the absence of significant findings in the P3 data.

This study offers three significant theoretical contributions. Firstly, it extends cue utilization theory from general consumer behavior to addiction-related decision-making. By examining how smoking-related cues influence smokers’ brand perception and WTP, the study provides new insights into how addiction reshapes decision-making processes. Secondly, the research bridges the gap between ERP findings and addiction studies. By linking the N1 and P3 components to attentional and motivational processes, it integrates neurophysiological perspectives with behavioral theories of addiction, enhancing the understanding of the neural mechanisms underlying cue reactivity in addiction. Lastly, the study emphasizes the role of social contexts in inhibitory control. The findings suggest that social environments may impair smokers’ ability to process and inhibit responses to smoking-related stimuli. This highlights the importance of incorporating social facilitation effects into the study of addictive behaviors, offering a more nuanced perspective on the interaction between social dynamics and inhibitory processes.

The practical implications of this study are as follows. Firstly, smoking control efforts should not only target action cues but also social cues. This can be achieved by enhancing anti-smoking education within social activities to help smokers control their smoking cravings and reduce smoking behavior. For instance, there could be restrictions on tobacco companies’ targeted advertising and marketing through social media channels to reduce the influence of smoking in social environments. Secondly, in social support and counseling services for smokers, it is essential to emphasize not only the health hazards to oneself but also the harm to others. Substance replacement smoking cessation programs should also incorporate alternative and supplementary social rewards. Social support can encompass online social groups, smoking cessation applications, and supportive and inspirational content on social media. These strategies aim to reduce the promoting effects of social marketing on smoking consumption behavior, enhance the regulation of tobacco advertising on social media platforms, provide smoking cessation support and education, and promote social rejection and resistance to smoking. By implementing these measures collectively, the impact of smoking consumption issues within the social environment can be reduced, thereby safeguarding public health and well-being.

Regarding the limitations of this study: (1) Although this study provides insights into the relationships between smoking-related cues, brand perception, and WTP, it does not establish causal connections. Future studies could utilize advanced statistical methods, such as regression, mediation analysis, or structural equation modeling, to better elucidate the causal pathways underlying these effects; (2) Although multiple brand images were used to minimize interference from brand awareness differences, the representativeness of the stimuli remains limited. Smokers across different age groups may vary in how they prioritize the social aspects of smoking, with individuals entering adulthood and the workforce potentially placing more emphasis on social functions than college student smokers; (3) The passive observation design employed in this study resulted in less prominent P3 amplitudes, which might also explain the lack of significant differences between conditions. Conducting follow-up studies that include an active response component (e.g., Go/NoGo tasks) can better elicit P3 and other ERP components associated with inhibitory control and salience detection. This would provide more robust evidence for the neural mechanisms underlying cue reactivity in smokers; (4) The role of e-cigarettes in smoking behavior might have amplified the influence of social factors, especially among younger individuals. The interactions between novel tobacco products and social cues warrant further investigation.

## 5 Conclusion

This study utilized electrophysiological method to investigate the effects of smoking action and social cues on smokers’ perception of cigarette brand. Based on cue utilization theory and brand perception literature, this study measured smokers’ brand perception and WTP under smoking social and action cue conditions. It established the connection from external stimuli to EEG responses, brand perception, and ultimately smoking behavior. The results showed that smokers still exhibit inhibitory control under the influence of action cues, but if social cues are present simultaneously, inhibitory control will be impaired. Furthermore, both types of cues enhance brand perception, ultimately resulting in a higher WTP cigarettes. In practical terms, the research findings can assist consumers in making informed decisions regarding cigarette consumption and help businesses balance brand promotion and anti-smoking efforts.

## Data Availability

The raw data supporting the conclusions of this article will be made available by the authors, without undue reservation.
